# Enhancing Human Glycoprotein Hormones Production in CHO Cells Using Heterologous Beta-Chain Signal Peptides

**DOI:** 10.1134/S1607672923700576

**Published:** 2023-12-19

**Authors:** M. V. Sinegubova, D. E. Kolesov, L. K. Dayanova, I. I. Vorobiev, N. A. Orlova

**Affiliations:** Federal Research Center “Fundamentals of Biotechnology,” Russian Academy of Sciences, Moscow, Russia

**Keywords:** glycoprotein hormones, signal peptides, Chinese hamster ovary cells, transient expression

## Abstract

We studied the influence of heterologous signal peptides in the β-chains of glycoprotein hormones on the biosynthesis of these hormones in a transiently transfected culture of Chinese hamster ovary cells CHO S. When the natural signal peptides of the β-chains were replaced with the heterologous signal peptide of human serum albumin, cell productivity was increased 2–2.5 times for human luteinizing hormone, human chorionic gonadotropin, and human thyroid-stimulating hormone, but not for human follicle-stimulating hormone. No significant increase in cell productivity was observed for human azurocidin signal peptide and human glycoprotein hormone α-chain signal peptide. The used approach allows quick assessing the effect of heterologous signal peptides on the biosynthesis of heterodimeric proteins of various classes.

The family of mammalian glycoprotein hormones includes several adenopituitary hormones, including thyroid-stimulating hormone (TSH), follicle-stimulating hormone (FSH), and luteinizing hormone (LH), which are used for medical purposes, and human chorionic gonadotropin (hCG), which is produced primarily by the placenta. All glycoprotein hormones consist of two glycosylated subunits—α (common for the entire family) and β (specific)—and exert their physiological effect only in the heterodimeric form.

The conversion of pre-α and pre-β subunits containing signal sequences into their mature forms involves two events: cleavage of the signal peptide (occurs cotranslationally [1]) and glycosylation (occurs both co- and post-translationally [2]). Hormone subunits undergo folding and assembly in the endoplasmic reticulum (ER). For LH, it was demonstrated directly that folding of the β subunit requires the presence in the endoplasmic reticulum of the α subunit, which functions as a chaperone [3]. It is very likely that all glycoprotein hormones in vivo undergo folding of β-chains in complex with the α-chain; however, it is not possible to strictly prove this statement for other hormones, because in vitro β-chains can be folded and secreted by cells independently of α-chain [4].

Since in modern clinical practice all glycoprotein hormones are used in recombinant form, increasing the specific productivity of the cells secreting them (primarily Chinese hamster ovary cells, CHO) remains an urgent task. One of the known rate-limiting stages of the classical protein secretion pathway is the co-translational translocation of synthesized polypeptides into ER. It was shown that various signal sequences can increase the rate of passage of this stage, which, in turn, leads to an increase in protein secretion [5]. Apparently, there is no universally effective signal peptide for proteins of different classes [6]. Moreover, it was shown in CHO cells that the native signal peptide is not necessarily the most effective [5, 7, 8]. In particular, Kober et al. showed an increase in the level of secretion by CHO cells of model proteins (antibodies and Fc fusion protein) with heterologous signal sequences—the prepro-protein of human serum albumin and the prepro-protein of azurocidin [9]. To increase the level of expression of recombinant proteins, non-natural (synthetic) signal peptides are also actively studied [10, 11]. Replacing native signal peptides with heterologous ones made it possible to increase the titer of recombinant antibodies [12]. However, for glycoprotein hormones this issue remains completely unexplored.

Previously, we discovered that, when FSH subunit genes are expressed in CHO cells as part of a tricistronic plasmid, the cells preferentially secrete the free α-subunit rather than the heterodimeric hormone [4]. The accumulation of large amounts of free α-subunit in the culture medium was stopped by repeated transfection of cells with a plasmid encoding the FSH β-subunit gene and an additional selection marker [4].

This procedure for balancing the levels of biosynthesis of hormone subunits requires too much time to obtain producer lines and, apparently, does not allow selecting the most productive cell clones due to the independent distribution of expression levels of hormone subunit genes in different individual cells. We assumed that the rates of biosynthesis of β-subunits of glycoprotein hormones can be reduced by their natural suboptimal signal peptides. In this case, replacing these peptides with more effective ones can lead to an increase in the level of biosynthesis of heterodimeric hormones.

To identify more effective signal peptides of hormone β-subunits, we used the p1.1-Tr2-Gon BIA plasmids, based on the p1.1-Tr2 vector plasmid developed by us previously [13, 14]. These plasmids encode the β-subunits of hormones in the first cistron of the tricistronic matrix, the α-subunit of hormones, which is the same in all cases, in the second cistron and the selection marker dihydrofolate reductase (DHFR) in the third cistron, i.e., the β-chain-IRES-α-chain-IRESatt-DHFR scheme (Fig. 1). For each β-chain, four different signal peptides were encoded: the native signal peptide of the β-chain of the corresponding hormone (NSP), as well as signal peptides heterologous to the β-chain (azurocidin (Azu)), human serum albumin (HSA), and α-chain (aSP), which is common for glycoprotein hormones.

**Fig. 1.**
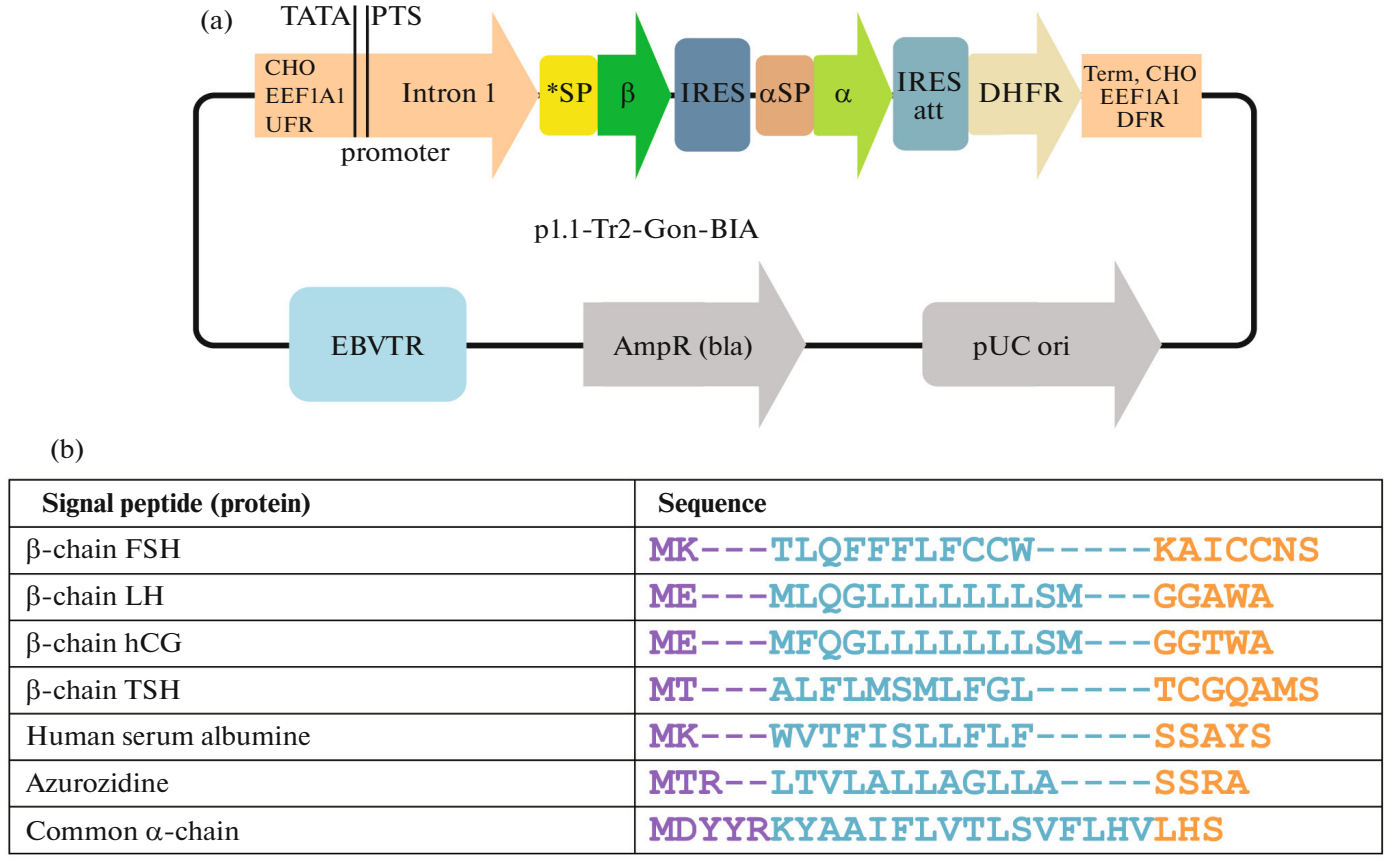
Tricistronic expression plasmids encoding glycoprotein hormone chains and signal peptide sequences. (a) Plasmid scheme, designations: pUC origin, region of origin of replication of the pUC plasmid; bla, beta-lactamase open reading frame; EBVTR, human Epstein–Barr virus terminal repeat region; CHO *EEF1A1* UFR and CHO *EEF1A1* DFR, regions flanking the Chinese hamster *EEF1A1* gene, contain the promoter, intron, terminator, and polyadenylation signal of the *EEF1A1* gene; TATA, TATA box; PTS, putative transcription start; IRES, natural internal ribosome entry site of wild-type EMCV; IRESatt, attenuated internal ribosome entry site; *SP, β-chain variable signal peptide; β, β-chain ORF; αSP, signal peptide of the glycoprotein hormone α-chain; α, α-chain ORF; DHFR, mouse dihydrofolate reductase ORF. (b) Sequences of signal peptides, the N-region is highlighted in purple according to the SignalP algorithm, the H-region is in blue, and the C-region is in orange.

Obtaining coding sequences with natural signal peptides for human FSH and LH is described in [4] and [15], respectively. In the case of hCG and TSH, we used synthetic β-chain genes, obtained by reverse translation of the NP_000728 and NP_000540.2 sequences, respectively. Synthetic “β-chain-IRES-α-chain” sequences were cloned into expression vectors using the *Abs*I–*Nhe*I restriction endonuclease sites. To replace the signal peptides of the β-chains, we performed step-out PCR using pairs of long adapter primers, which encoded the signal peptide and the synthetic Kozak sequence, and a reverse primer common for each β-chain. PCR products were subcloned into a T vector, sequenced, and transferred into expression plasmids at the *Abs*I-*Spe*I/*Nhe*I sites, replacing the native β-chain.

The probability of correct processing of all selected signal peptides in combination with the corresponding subunits, calculated using the SignalP 6.0 bioinformatics service [16], was more than 95% in all cases. Thus, all 16 plasmids were expected to produce heterodimeric hormones in the culture medium after transfection of CHO cells. The obtained plasmids were mixed in phosphate-buffered saline (ratio 95 : 5) with the control plasmid encoding the green fluorescent protein eGFP (pEGFP-N2, Clonentech, United States, Addgene #6081-1) and transfected into CHO S cells using the GenJect39 liposomal reagent (Molecta, Russia); transfection was performed in three biological replicates. Then, 3 days after transfection, the concentrations of heterodimeric hormones in the culture medium were measured by ELISA. The following antibodies were used: conjugate of monoclonal antibodies against the α-subunit of glycoprotein hormones with horseradish peroxidase #XF1*, monoclonal antibodies against the LH β-subunit #XL1, monoclonal antibodies against the FSH β-subunit #XF2, monoclonal antibodies against the TSH β-subunit #XTB1, and monoclonal antibodies against the hCG β-subunit #XH51; all antibodies were from XEMA LLC, Russia). We also determined the total concentrations of α-subunits in the culture medium. The following antibodies were used: conjugate of the monoclonal antibody against the α-subunit of glycoprotein hormones with horseradish peroxidase #K003/1* and monoclonal antibodies against the α-subunit of glycoprotein hormones with horseradish peroxidase #K003; all from Diatekh LLC, Russia. Cells were also collected and lysed, and the eGFP fluorescence intensities in the lysate were measured to normalize the data. For all constructs, secretion of heterodimeric forms of hormones into the culture medium was observed (Fig. 2).

**Fig. 2.**
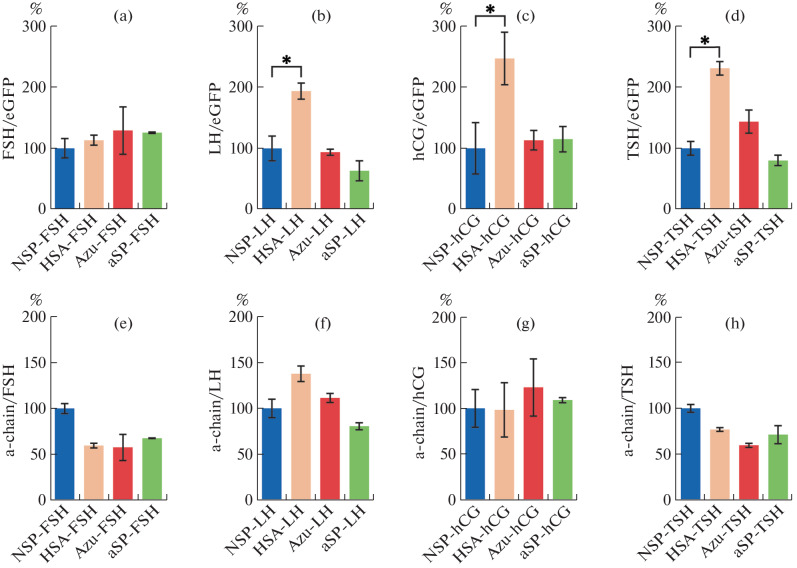
Titer of glycoprotein hormones secreted by a transiently transfected CHO cell culture when varying the signal peptides of their β-chains. (a–d) Titer of the heterodimeric form of the hormone (assessed by ELISA) normalized to the level of the control green fluorescent protein eGFP (assessed by fluorimetry). (e–h) Titer of the secreted α-chain with the native signal peptide (assessed by ELISA) normalized to the titer of the heterodimeric form of the hormone. Data are represented as the mean value of three independent biological replicates, with two ELISA measurements for each sample; the value for the control peptide NSP is taken as 100%. * *p* < 0.05, Tukey’s test. Abbreviations: FSH, follicle-stimulating hormone; LH, luteinizing hormone; hCG, human chorionic gonadotropin; TSH, thyroid-stimulating hormone; NSP, native signal peptide of the corresponding glycoprotein hormone β-chain; HSA, human serum albumin; Azu, azurocidin; aSP, signal peptide of the glycoprotein hormone α-chain.

For all hormones except FSH, when the native signal peptides of the β-chains was replaced with the heterologous HSA signal peptide, a statistically significant (2–2.5 times) increase in the level of secretion of heterodimeric hormones was observed. At the same time, for FSH and TSH, but not for LH and hCG, a decrease in the relative level of secretion of all forms of the α-subunit was recorded when the native signal peptides of the β-subunit were replaced with the heterologous ones. We assume that, for the LH and hCG hormones, which have highly homologous β-subunits, the free α-subunit is retained in the ER before binding to the folded β-subunit. With an increase in the number of β-subunits in the ER, an increase in the levels of secretion of heterodimeric hormones, but not a drop in the levels of secretion of free α-subunits, can be observed.

The approach used in this study demonstrates that, for the majority of glycoprotein hormones, cell productivity can be increased by increasing the efficiency of translation and translocation of hormone β-subunits into the ER by combining them in a frame with heterologous signal peptides, among which the most significant increase in productivity is ensured by the HSA signal peptide. The data obtained by transient transfection can be further refined for stably transfected cell populations with higher specific cell productivity.
